# Artificial Intelligence in Malnutrition: A Systematic Literature Review

**DOI:** 10.1016/j.advnut.2024.100264

**Published:** 2024-07-04

**Authors:** Sander MW Janssen, Yamine Bouzembrak, Bedir Tekinerdogan

**Affiliations:** Information Technology Group, Wageningen University and Research, Wageningen, The Netherlands

**Keywords:** machine learning, decision support, malnutrition, nutritional assessment, nutritional screening tool, personalized nutrition, precision nutrition

## Abstract

Malnutrition among the population of the world is a frequent yet underdiagnosed problem in both children and adults. Development of malnutrition screening and diagnostic tools for early detection of malnutrition is necessary to prevent long-term complications to patients’ health and well-being. Most of these tools are based on predefined questionnaires and consensus guidelines. The use of artificial intelligence (AI) allows for automated tools to detect malnutrition in an earlier stage to prevent long-term consequences. In this study, a systematic literature review was carried out with the goal of providing detailed information on what patient groups, screening tools, machine learning algorithms, data types, and variables are being used, as well as the current limitations and implementation stage of these AI-based tools. The results showed that a staggering majority exceeding 90% of all AI models go unused in day-to-day clinical practice. Furthermore, supervised learning models seemed to be the most popular type of learning. Alongside this, disease-related malnutrition was the most common category of malnutrition found in the analysis of all primary studies. This research provides a resource for researchers to identify directions for their research on the use of AI in malnutrition.

**Statement of Significance**

This study highlights the critical gap between the development and clinical implementation of artificial intelligence tools for malnutrition detection, advocating for their adoption to enhance early diagnosis and patient outcomes.

## Introduction

The WHO has declared healthy aging a priority of its work on aging between 2016 and 2030 and has come up with a global strategy and action plan related to aging and health from 2016 to 2030 [1]. Malnutrition is a critical and global concern that requires continuous intervention strategy.

Currently, 1 of 10 people is older than 65 y; the WHO has estimated this number to be 1 of 6 in 2050. Malnutrition is an increasing health problem in people older than 65 y even in developed countries and in nursing homes, hospitals, and acute care units [2]. With this ever-increasing number of elderly population throughout the world, the potential number of patients with malnutrition will see a significant increase further highlighting the emphasis of the problem [3].

Not only adults but also many children in the world experience malnutrition. Malnutrition in children can be classified as stunting (i.e., too short for their age), wasting (i.e., too thin for their age), and overweight (i.e., too heavy for their age). The WHO published updated prevalence numbers in 2023: 148 million children younger than 5 y in the world experience stunting, 45 million wasting, and 37 million overweight globally [4]. These numbers reflect the need for interventions worldwide.

Malnutrition is a multifaceted concept that can be seen as an umbrella term that serves as an overarching theme for understanding various nutritional conditions and their associated physiologic consequences. Recent efforts from the European Society for Clinical Nutrition and Metabolism have been made to come up with a definition. Malnutrition can be defined as “a state resulting from lack of intake or uptake of nutrition that leads to altered body composition (decreased fat free mass) and body cell mass leading to diminished physical and mental function and impaired clinical outcome from disease”[5].

**FIGURE 1:**
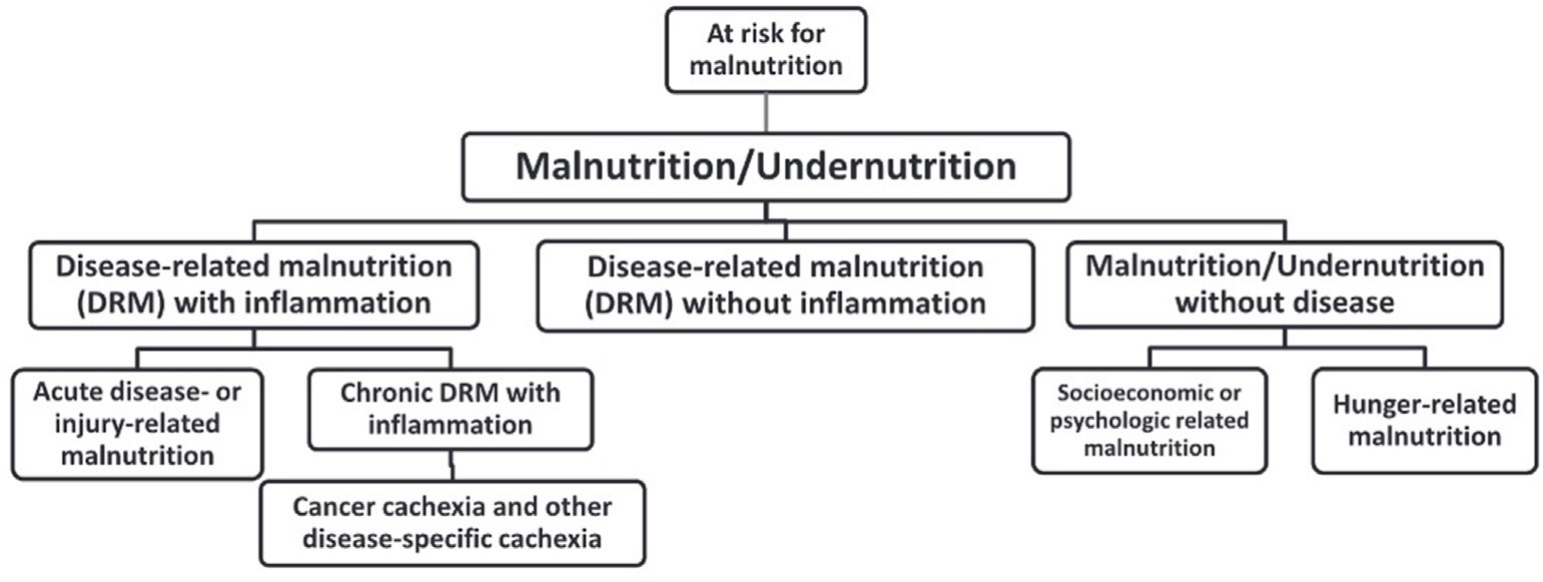
The malnutrition taxonomy [5].

Owing to the complex nature of malnutrition, it can be hard to distinguish between other conditions that seem very similar on the surface, such as cachexia or sarcopenia. A detailed taxonomy is depicted in Figure 1 [5]. Furthermore, different screening tools are used: for example, the Malnutrition Universal Screening Tool (MUST) [6], Mini Nutritional Assessment [7], Patient-Generated Subjective Global Assessment (PG-SGA) [8], and WHO z-scores [9]. Many of these tools are patient group specific and therefore lack generalizability. To date, no 1 golden standard for diagnosing malnutrition exists [10]. Because of this issue, international efforts have been made by Global Leadership Initiative on Malnutrition (GLIM) to come up with diagnostic criteria that may be used by health care professionals globally to diagnose malnutrition in patients [11].

Furthermore, the WHO has created standard weight-for-age, weight-for-height, and height-for-age scores. These are used alongside BMI standards to determine whether a child is malnourished [9]. Both initiatives aim to provide a standardized framework for diagnosing malnutrition.

However, the treatment of malnutrition is a multidisciplinary effort, therefore it can be taxing on the health care system. Because of this, many cases of malnutrition go unnoticed, which leads to further complication, increasing the economic impact, productivity, and prosperity [12,13].

Recent advancements in artificial intelligence (AI) use aid in performing some of the tasks. One technique that has recently benefited from AI enhancements is decision support systems (DSS), which assist professionals in making complex decisions [14]. This brought rise to the clinical decision support systems, which have proven to be useful but yet to reach widespread implementation [15]. These may be used by health care professionals to aid them in the decision-making process on treatment options or diagnosis (e.g., to treat malnutrition or cancer). The distinction being in the specificity. Clinical decision support systems are tailored for use within the medical setting, compared with DSS that are more general and encompass various decision-assisting frameworks. In this article, both will be referred to as DSS for readability purposes; nonetheless, it is of importance for the reader to understand the aforementioned difference.

The field of medicine has been using both simple and more complex DSS in the past decade. However, in the field of clinical nutrition, DSS research has been lagging. Modern studies show improvements in both diagnostic and screening tools compared with traditional methods. Although the DSS progress related to clinical nutrition has been slow, it has been proven to be very promising [16,17].

Further research into the topic of DSS in malnutrition is required so that these systems can be implemented in practice. Despite our best efforts, we were unable to find a systematic literature review (SLR) on DSS use in malnutrition. This study represents a pioneering effort that paves the way for systematically reviewing the latest knowledge on DSS in the field of clinical nutrition related to malnutrition. In this article, an SLR was conducted with the aim to discover and compile existing research on DSS and give a comprehensive and complete overview of all research to date.

## Review Protocol

**FIGURE 2:**
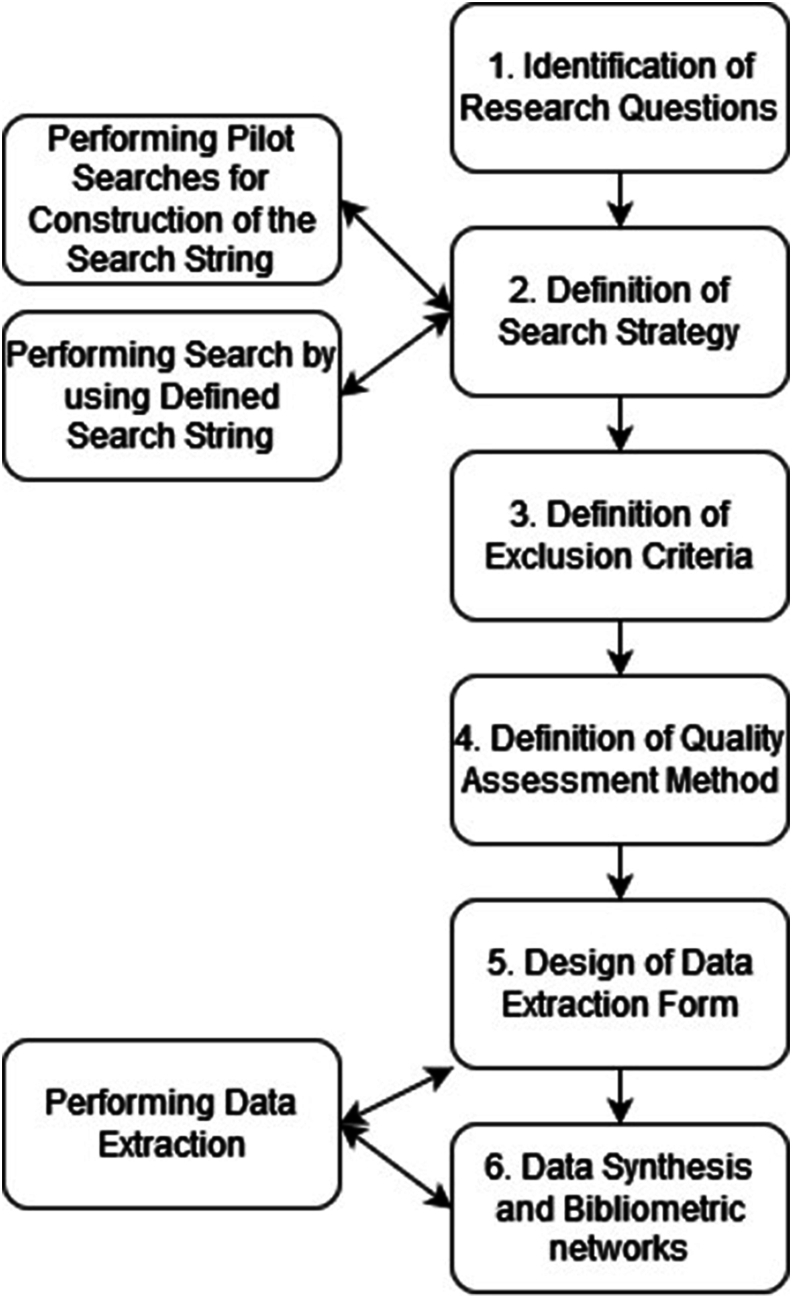
Review protocol for this systematic literature review based on the study by Kitchenham et al. [18].

The guidelines used in this SLR are the ones created by Kitchenham and Charters [18]. These are guidelines created for conducting SLRs in the field of software engineering adapted from guidelines based on SLRs in the medical field. As the topic of this article extends over both fields, these guidelines are a reasonable choice to use. Using these guidelines [15], a protocol was carefully constructed, which is depicted in Figure 2 [18]. The initial phase involved formulating research questions, which guided the development of our search strategy and the resulting search terms in step 2. Alongside the creation of the search query, a pilot search was conducted with the aim of identifying relevant keywords. Furthermore, step 2 included the identification of relevant digital libraries. The definition of the search query was an iterative process, where the output of 1 search query was used to alter the subsequent search query. In step 3, we established specific criteria for selecting relevant studies. Subsequently in the fourth step, we applied these criteria to the search results, resulting in a collection of relevant studies. These studies were then judged using the quality assessment method. Step 5 was the creation of a data extraction from that allowed useful data extraction from the selected articles. The third, fourth, and fifth steps were iterative. In the sixth step, we outlined techniques for data synthesis and presentation of the data. Ultimately, in the seventh and final step, the bibliometric networks and their use case were discussed.

### Research questions

The following 4 research questions were derived to which the SLR should propose a solution:

• Q1: What type of manual malnutrition tool is being used?
  ○ Setting, patient group

Malnutrition tools relate to specific patient groups and settings in which they are used. This research question is aimed at exploring the most prevalent tools and settings.

• Q2: Which machine learning (ML) techniques have been used in these studies?
  ○ Q2.1: Supervised, unsupervised, or semisupervized or reinforcement learning?
    ⁃ What type of algorithms are being used?
  ○ Q2.2: What problems was the algorithm assigned to?
• Q3: What is the stage of implementation?

The stage of implementation is of importance to gain a deeper understanding of how many proposed solutions are used in day-to-day practice. Exploring the stage of implementation will help to quantify this.

• Q4: What are the challenges when using DSS in malnutrition?

### Search strategy and selection criteria

To answer the research questions, an SLR was conducted. The following digital libraries were used: PubMed, Scopus, Google Scholar, and Web of Science. This selection was based on expert knowledge in the field of nutrition and data science. The field of malnutrition and ML is rapidly advancing so all articles that were published over 10 y ago were excluded from this review. Furthermore, the targeted sources were journal articles, conference articles, and white articles. The automated search was done using a search query. Every syntax was altered to suit different libraries but, in general, the query looked like this:

“(Malnutrition OR Undernutrition) AND (Artificial Intelligence OR Machine Learning) AND (tool∗ OR App∗)”

The full list of queries sorted per library can be found in Supplemental Table 1. Next to the automated search through the previously described libraries, a manual search was executed. The manual search was done by going through the references of the articles that were found by the search string. We looked at both the sources the authors referenced and the ones they were referenced by and included all relevant articles. The results of the search query are presented in Table 1. The order of the libraries in the articles is based on the order of use. Naturally, the number of useful articles reduced owing to the duplicate articles that were found. In total, 330 articles were identified after the automated search and 4 with the manual search, totaling to 334.

**TABLE 1:** Overview of search results and study selection

Source	After automated and manual search	After applying selection criteria	After reading complete study and quality assessment
PubMed	24	13	10
Scopus	174	39	27
Google Scholar	21	2	2
Web of Science	111	10	6
Manual Search	4	4	4
Total	334	68	49

### Study selection criteria

To avoid overlooking any pertinent articles, a broad search query was used, resulting in a substantial number of articles, from which we filtered the most relevant ones using the selection criteria presented in Table 2. Initially, each article was individually assessed by reviewing its title and abstract to check for relevance. This brought down the number to 68 articles (Table 1). Next, the articles were read in their entirety and the selection criteria applied again. This lowered the total number of included articles to 49 (Table 1).

**TABLE 2:** Study selection criteria

No.	Criterion
EC1	Articles without full-text available
EC2	Articles not written in English
EC3	Articles not in line with research question
EC4	Articles that used manual tools
EC5	Articles that are experience and survey articles
EC6	Duplicate publication from multiple sources

### Study quality assessment

**FIGURE 3:**
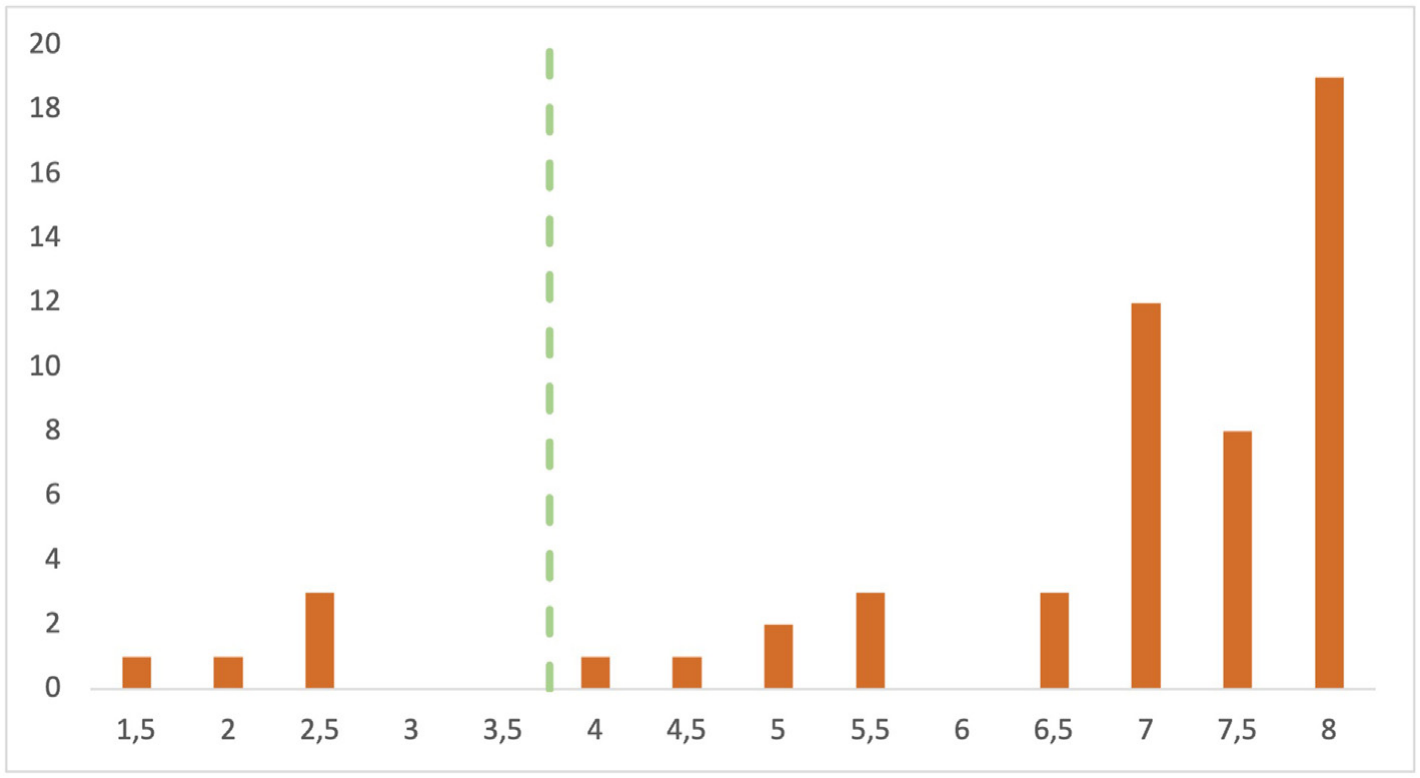
Primary study quality assessment scores. The line indicates the cutoff value to maintain high quality for this systematic literature review.

Following the inclusion of the 49 articles, the assessment of quality was done using the assessment quality criteria. These quality criteria are based on a previous study [18]. The quality assessment was executed by reading the entire article and applying the quality criteria presented in Table 3. The quality assessment was based on a score system. If answered yes, the article was rewarded with 1 point; if answered partially, the article was rewarded with 0.5 points; and if the answer is no, the article was rewarded with 0 points. A total of 8 points can be given to an article if all questions are answered with yes, rewarding 1 point each. For example, Q7 (Are the negative findings presented?), when reading the article, we looked for the limitations at the usual place (discussion section of the article). If they were present, 1 point was awarded. If the authors mentioned limitations but not at the usual place or the limitations were poorly described, 0.5 points were awarded, and if the limitations were not mentioned at all, 0 points were awarded. The decision to exclude all articles with a quality score lower than 4 was made to ensure high-quality input of primary studies for this SLR. Five low-quality articles were excluded, resulting in 49 articles that were included in the final SLR. The distribution of the study quality assessment score of all articles can be found in Figure 3.

**TABLE 3:** The quality assessment criteria presented by Kitchenham et al. [18] as a means of assessing article quality

No.	Questions
Q1	Are the aims of the study clearly stated?
Q2	Are the scope and context of the study clearly stated?
Q3	Is the proposed solution clearly explained and validated by an empirical study?
Q4	Are the variables used in the study likely to be valid and reliable?
Q5	Is the research process documented adequately?
Q6	Are all study questions answered?
Q7	Are the negative findings presented?
Q8	Are the main findings stated clearly in terms of creditability, validity, and reliability?

### Data extraction

The 49 articles were read in their entirety and the required data extracted. To efficiently store all data, a data extraction form was created in Microsoft Excel. We first read 3 randomly selected articles and defined the initial variables in the extraction form on this. Next, 5 additional articles were randomly selected from which we extracted the data and added any new important variables. Iterations of this process were executed until all data from all studies was present in the data extraction form. This form contains 26 elements, which cover but are not limited to basic information like the author names, date of extraction, library it was extracted from, and title (Table 4). Next to this, other information was extracted such as the type of algorithm, type of malnutrition, traditional tools used, and identified problems (Table 4). We used the extraction form to pinpoint the most used algorithms and malnutrition diagnostic tools in the literature.

**TABLE 4:** Data extraction form

Information extracted from the articles		
ID Title Downloaded: yes/no Date of extraction Year of publication Author name(s) Repository Type	Quality assessment Type of malnutrition Type of patient group Setting Type of machine learning Based on what original tool Type of algorithm Purpose of created tool	Software used Implementation stage Goal of model Country of origin Variables used for input Reason for writing Challenges presented Proposed solution

### Data synthesis and bibliometric networks

The data synthesis and visualization were the last part of the review process. The goal was to define common theme under umbrella terms to digest the information and make it viable to analyze the data. The data from step 5 were used as input. For example, malnutrition, being a very broad term, was divided into 3 big categories: disease-related malnutrition, non–disease-related malnutrition, and children, the latter referring to malnutrition research in children (younger than 18 y). This allowed very simple visualization of the different domains in an easy and understandable way without losing out any valuable information. Next, we synthesized any synonyms present. For example, any variation of height-for-weight z-scores used in malnutrition related to children was combined into WHO z-scores for ease of finding a trend in the data.

Furthermore, the included primary studies were further analyzed using the bibliometric network methods such as citations, keywords, and co-authorship analysis as demonstrated previously [[19], [20], [21]]. Overlay visualizations were built to visualize the bibliometric networks using the open access tool named VOSviewer^1^. The VOSviewer tool allows for bibliometric networks to be created ranging from citation relations between publications to the relationship between keywords of all included primary studies like has been showcased by a previous study [22]. Overlay visualization makes use of colored nodes. Each node indicates a certain property of said node. For instance, a node may represent a used keyword or author of a primary study. The size indicates the prevalence of this keyword or author. The color is used to create clusters of nodes [23,24].

## Results

In this section, the general findings regarding the 49 primary articles will be discussed. The rest of the section is dedicated to presenting the results for each research question in a chronologic order.

### Number of publications per year and country

**FIGURE 4:**
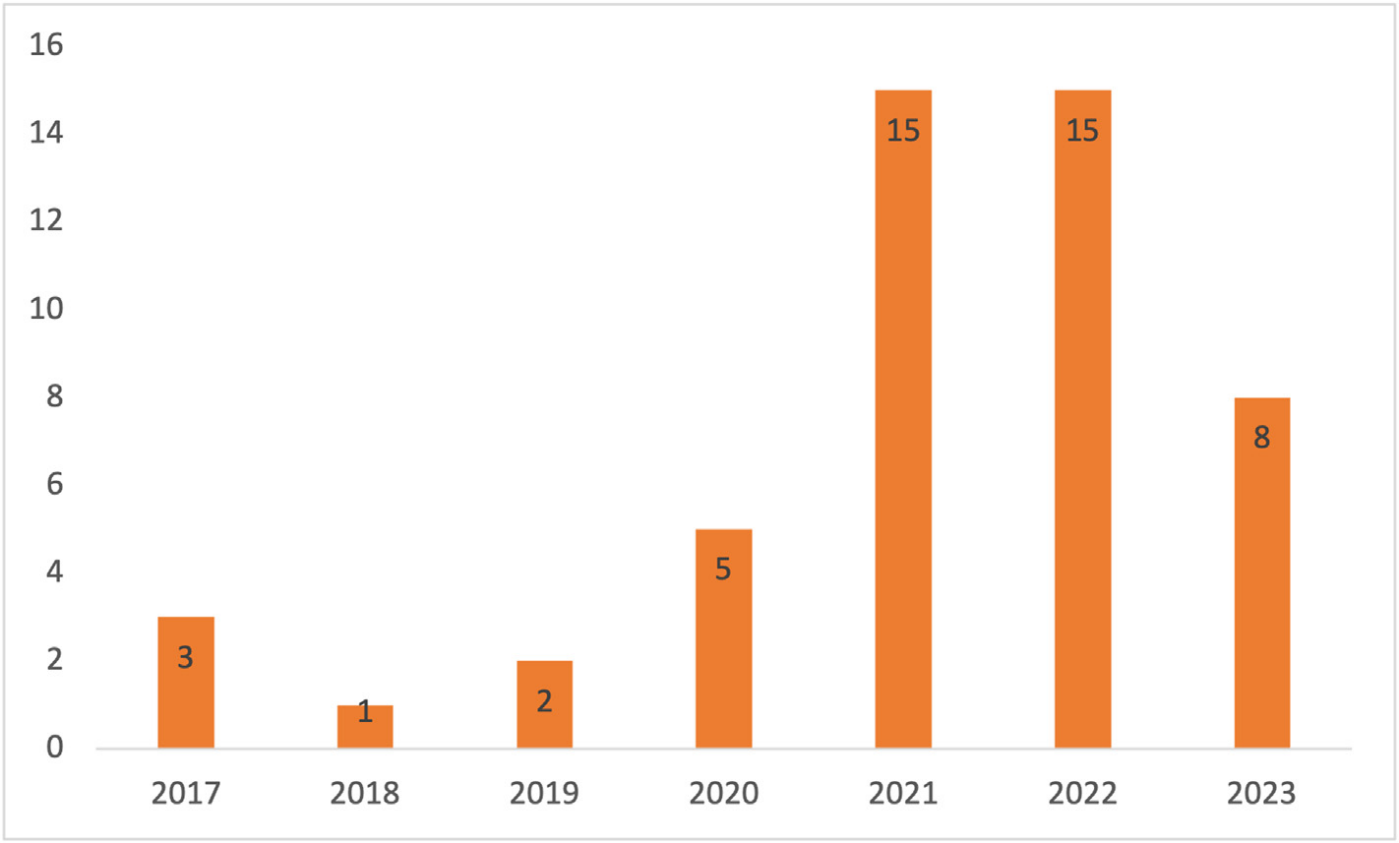
Year-wise distribution of the primary studies.

**FIGURE 5:**
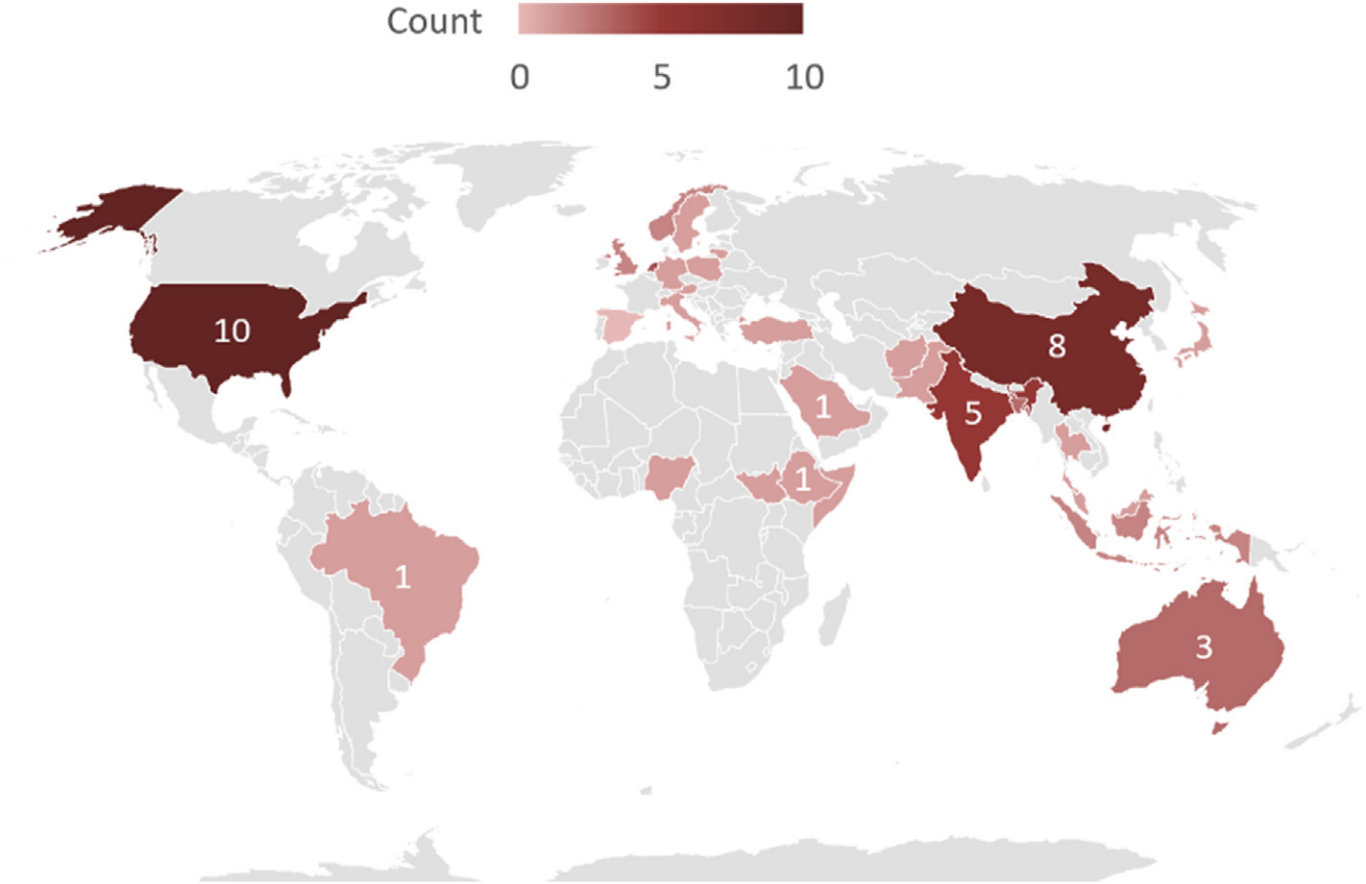
Distribution of primary studies per country.

The 49 primary studies are listed in Supplemental Table 2. The most popular digital library was Scopus with 174 hits converting to a final total of 39 including primary studies, which can be seen in Table 1. The most popular publication channel is PLoS One with 6 publications. It is noteworthy that most of the journals only occurred once in the list of publication channels, suggesting there is no one specific channel that is the best source for primary studies. The year-wise distribution can be seen in Figure 4, which shows that the most articles were published in 2021 and 2022 with a combined total of 30. Figure 5 shows the distribution of studies done by country. Notably, most studies (10 studies) were conducted in the United States, which is a significant portion of all studies in this SLR.

**FIGURE 6:**
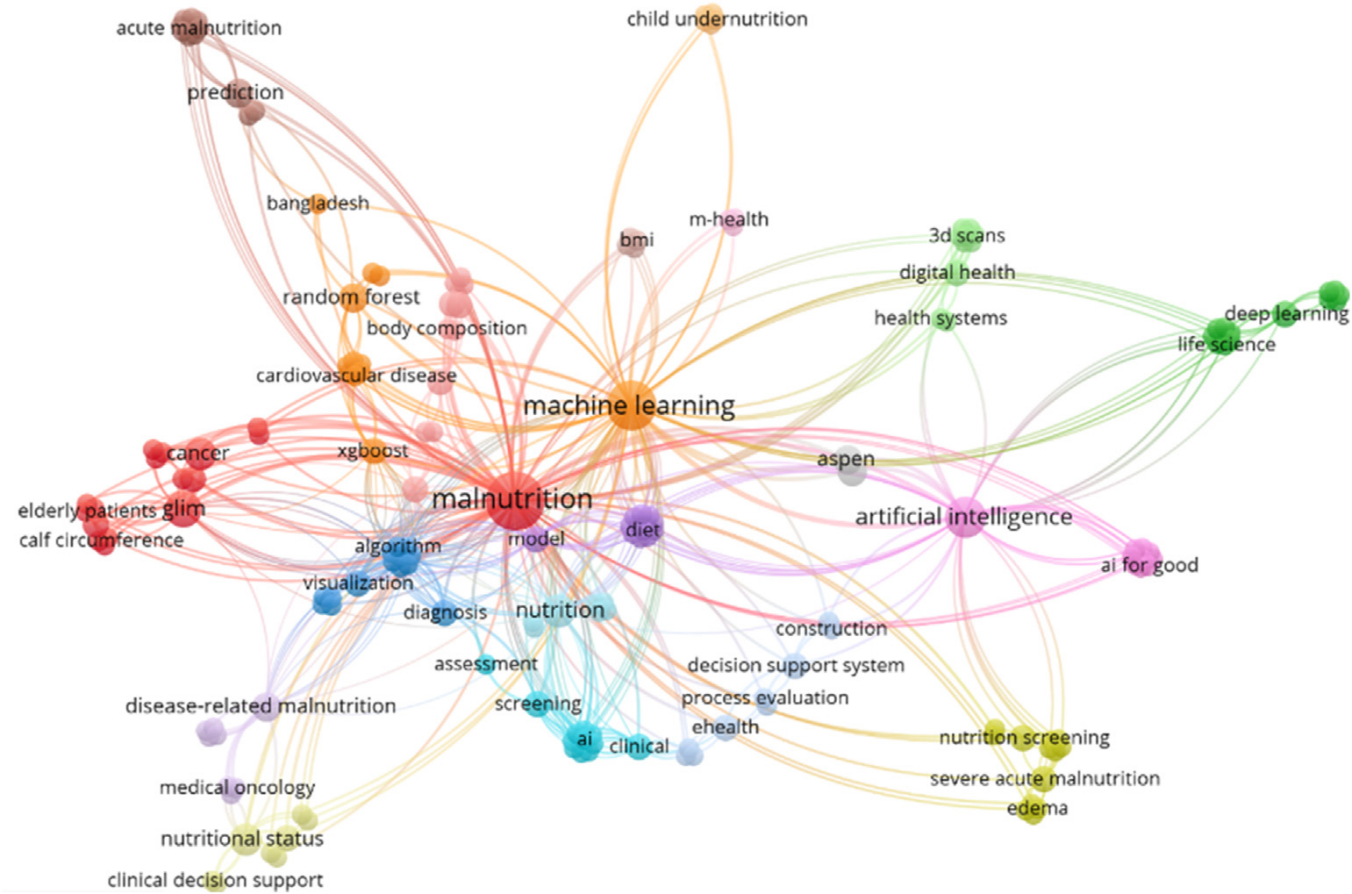
Network visualization of the keywords used in the included primary studies.

Figure 6 shows the network visualization of the keywords used based on all included primary studies. This visualization was created using VOSviewer. Each node represents a single keyword that was used. The size of the circle is related to the prevalence of each keyword. The co-occurrence of the keywords is denoted by the length of the lines. The nodes that are closer together indicate that these keywords often occur in combination with one another. This indicates that these nodes are correlated. In general, the closer the keywords are located together in the visualization, the more strongly they are related to one another based on bibliographic coupling. The color of each node indicates their determined cluster. In the visualization in FIGURE 6, FIGURE 11 clusters were identified. These consist of malnutrition-related indicators (in red), malnutrition prediction (in brown), algorithm related (in blue), diet (in purple), DSS (in light blue), clinical assessment (in turquoise), AI (in pink), ASPEN (in gray), ML (in orange), system solutions (in green), and body composition (in light red).

**FIGURE 7:**
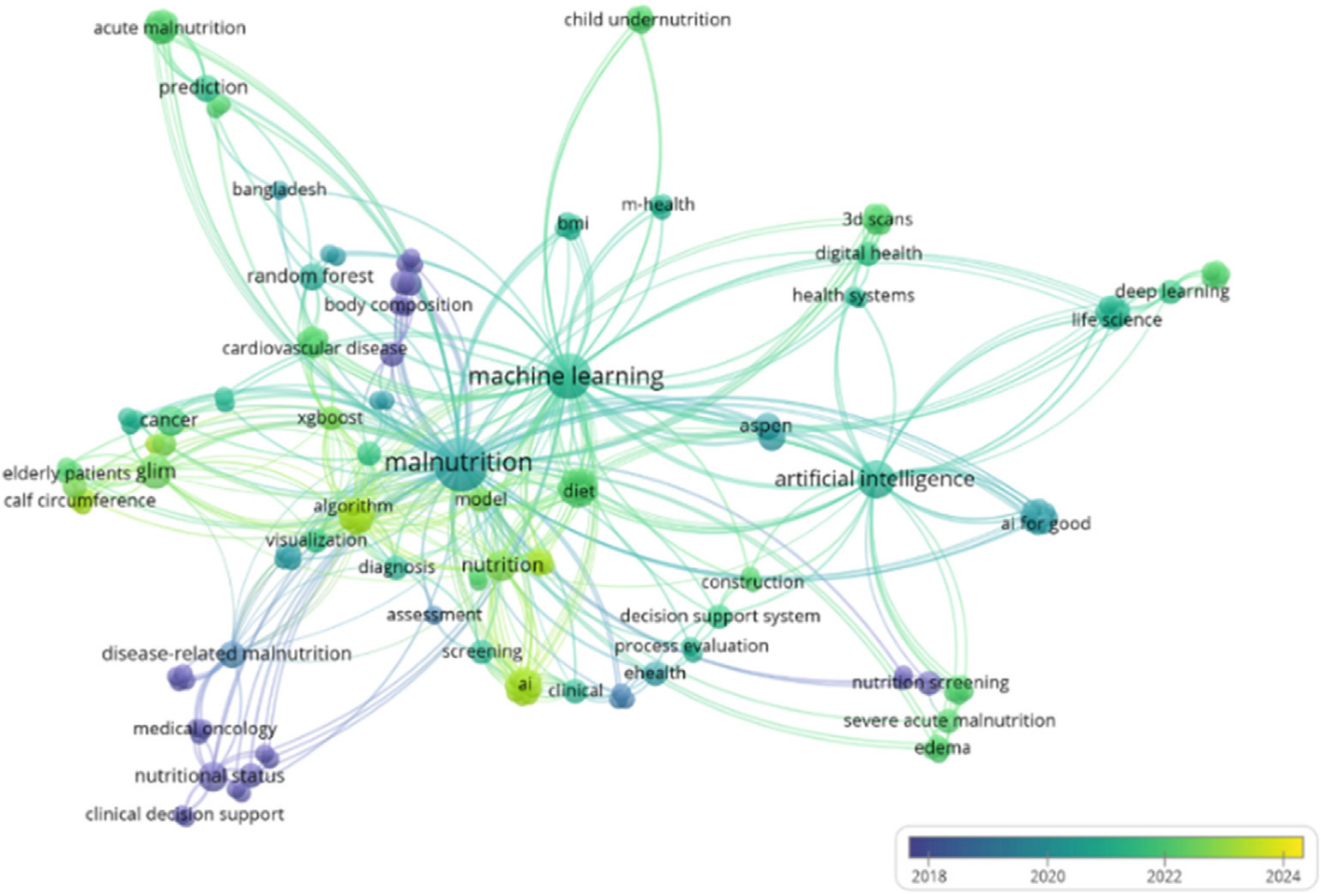
Network visualization of keywords sorted per year of primary study publication.

Next to this, another visualization for the keywords was created. The VOSviewer tool allows for sorting per year of publication. Figure 4 shows the distribution of articles published per year. The same keywords that were used in the network visualization of Figure 6 were used as input for the visualization of Figure 7. This allowed for the identification of publication topic trends. For instance, Figure 7 shows that most research in 2018 was done on nutritional states and clinical decision support (in blue). In the following years, this shifted toward malnutrition (in turquoise) in a broader scheme and finally to algorithms and AI (in yellow).

### RQ 1: What type of malnutrition tool is being used?

The current way to classify malnutrition is using some sort of guidelines tools or so-called questionnaires depending on the setting. In hospitals, it is common practice to make use of these questionnaires that consist of questions relating to variables or determinants of malnutrition in practice. This does not mean that patients get to send these questionnaires but rather a trained health care professional like a nurse or registered dietician uses these to assess their patients.

There is an important distinction between the screening questionnaire and the diagnostic questionnaire. The first is used to screen for existing risk of developing malnutrition, the latter is used to diagnose a patient with malnutrition. In practice, all patients who get admitted to a hospital are screened for their initial risk after which if risk hits some sort of threshold value, a trained professional, mostly a dietician, is then called upon to do the diagnosis using the diagnostic questionnaires like GLIM [11].

For example, MUST [25] may be used to assess initial risk of malnutrition in hospitals; subsequently, the GLIM criteria may be used to diagnose the type of malnutrition [26]. Notably, not a single article discussed any screening tools for child malnutrition although these do exist.

**FIGURE 8:**
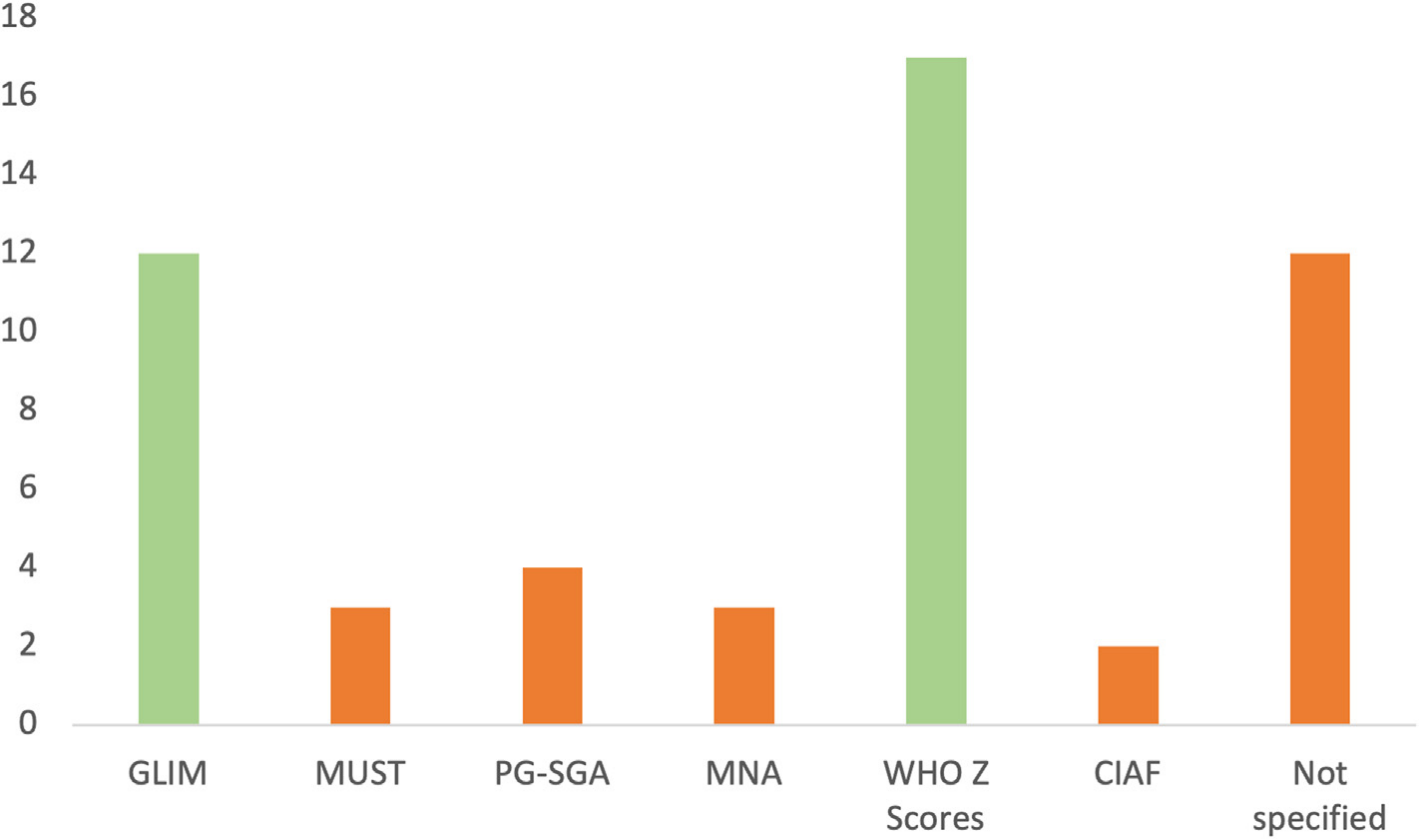
Type of manual tool used: diagnostic tools: Global Leadership Initiative on Malnutrition (GLIM), WHO; screening tools: Malnutrition Universal Screening Tool (MUST), Patient-Generated Subjective Global Assessment (PG-SGA), Mini Nutritional Assessment (MNA), and Composite Index of Anthropometric Failure (CIAF).

In Figure 8, the type of malnutrition screening and diagnostic tools is depicted. The most used diagnostic tool was the WHO z-scores, with a total of 17. The most used screening tools were MUST and Patient-Generated Subjective Global Assessment [26], both having a total of 4. However, 17 articles did not specify any screening or diagnostic tool use; this is very interesting given the fact that these are needed to accurately assess a person’s nutritional status. Without these, it would be hard to formulate a good diagnostic model let alone a DSS that aids in the screening and diagnostics of malnutrition.

#### Common patient groups and research setting

**FIGURE 9:**
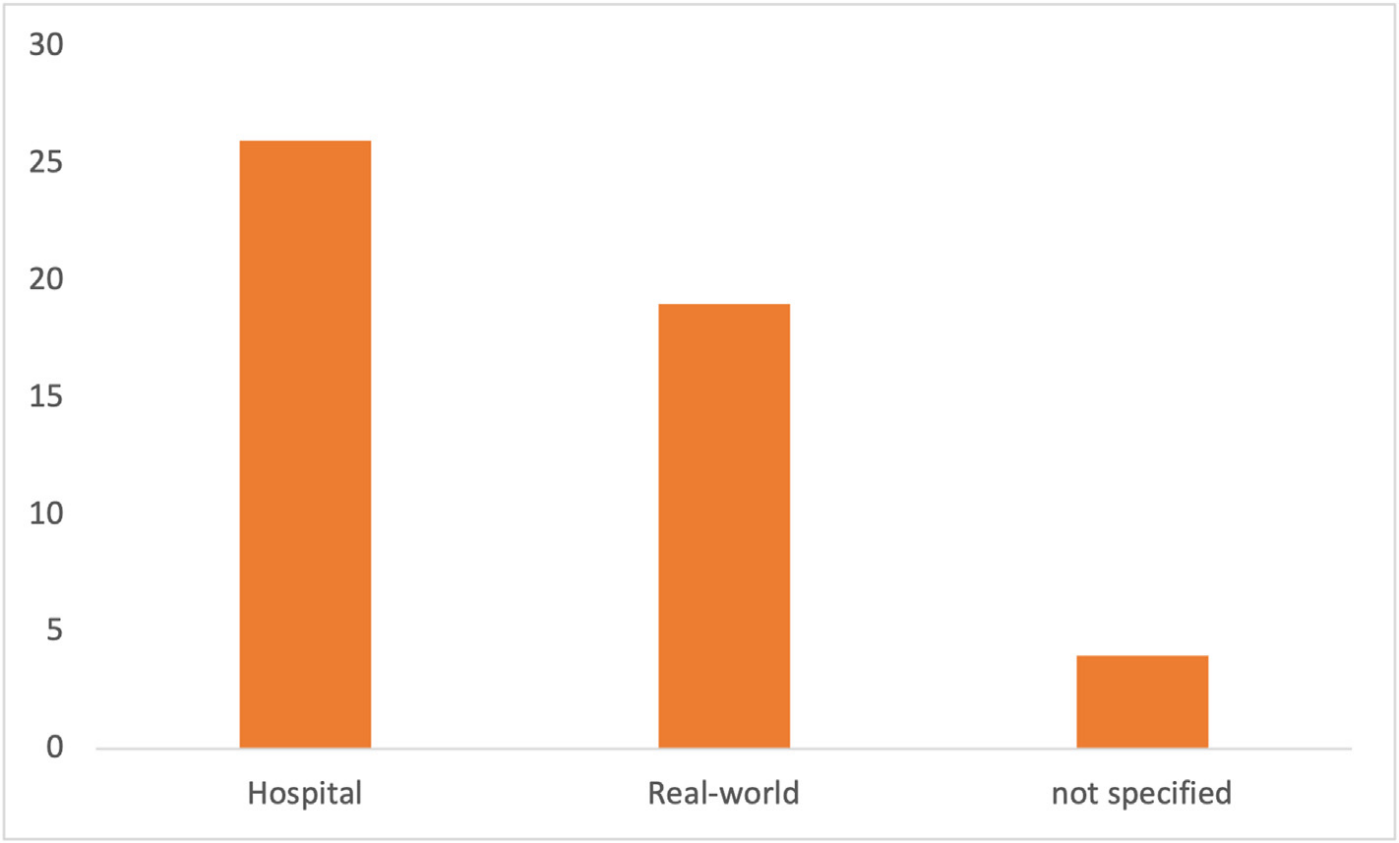
Related setting count.

**FIGURE 10:**
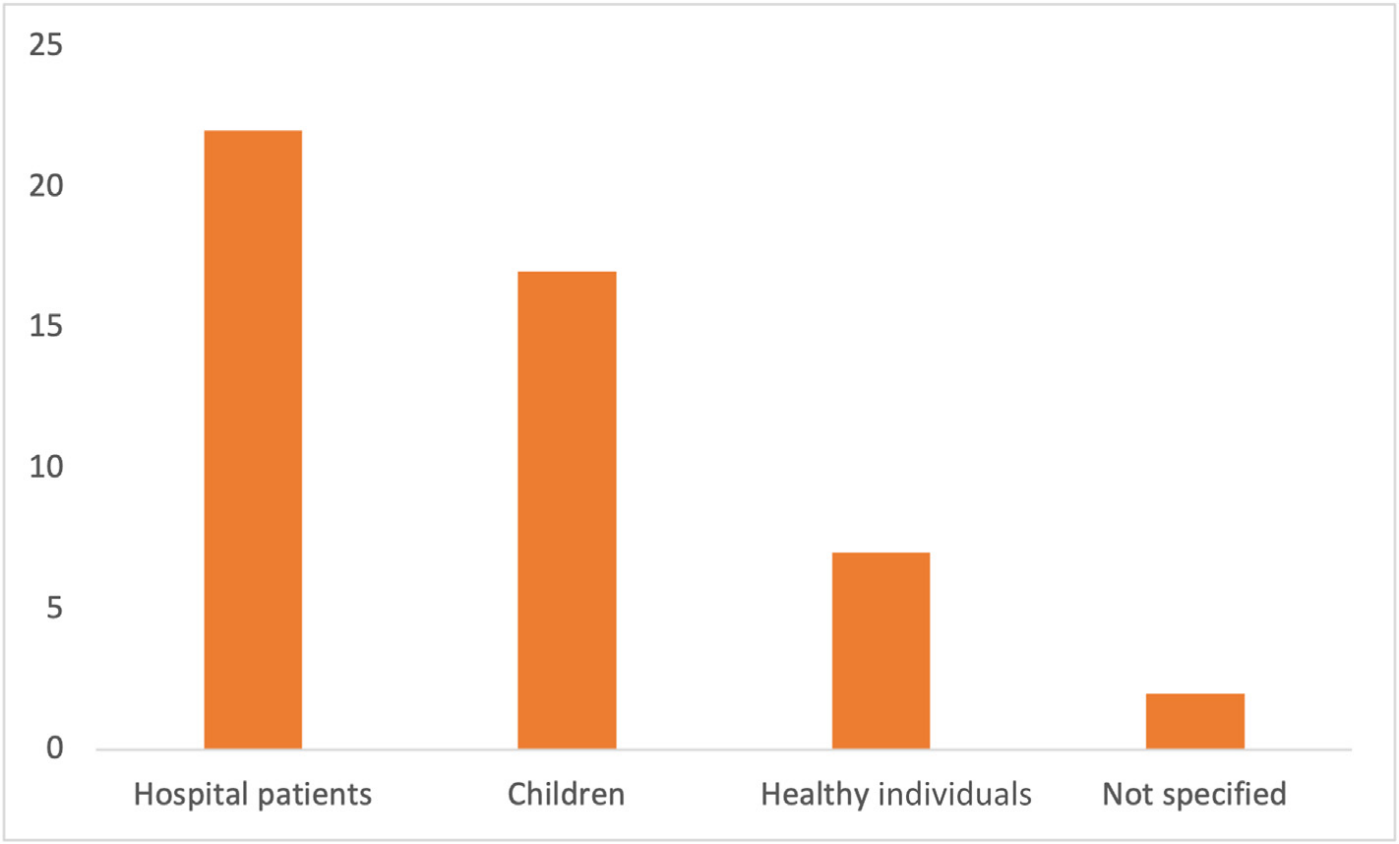
Type of patient group count.

Because these tools are setting and patient group specific, the most common setting and patient group are discussed next. The most common setting is depicted in Figure 9, with 26 articles in the hospital setting. The most common patient group being hospital patients, with a total of 22 article (Figure 10). Four articles did not specify their setting, or this information could not be extracted from the text. Real-world meaning any primary study that covered research that was conducted without a specific setting (e.g., country data, regional data, or free-living individuals). Interestingly, one can see that the real-world setting is closely related to the number of articles mentioning research into child malnutrition. This is because much child-related research was conducted using country-specific data or regional data. Only a handful of studies investigated hospitalized children. Adversely, most articles covering the topic of disease-related malnutrition were done in institutionalized adults.

### RQ 2: Which ML techniques have been used in these studies?

There are 4 general types of learning: supervised, unsupervised, semisupervized, and reinforcement learning. Supervised learning is characterized by the data having available labels, so the predicted value or class is known. This means the outcome may be verified against the truth. Unsupervised learning is the opposite where data occur without any labels at all, and the algorithm aims to find patterns in the data or partition them based on similarity. Semisupervized learning can be seen as a bridge between the latter 2. These algorithms make use of a subset of data being labeled while a big portion of the data are not. This is used to speed up the labeling part and improve accuracy of the algorithm. Finally, reinforcement learning is where a so-called agent exists in a dynamic environment and is penalized or rewarded for the decisions that it makes within this environment. The algorithm will be updated after each iteration to either maximize the potential reward or minimize the penalization or both at the same time [27].

#### Supervised, unsupervised, semisupervized, or reinforcement learning?

Table 5 [[25], [26], [27], [28], [29], [30], [31], [32], [33], [34], [35], [36], [37], [38], [39], [40], [41], [42], [43], [44], [45], [46], [47], [48], [49], [50], [51], [52], [53], [54], [55], [56], [57], [58], [59], [60], [61], [62], [63], [64], [65], [66], [67], [68], [69], [70], [71], [72], [73]] displays the amount of the different types of learning that have been used and their corresponding most used algorithm. It is important to note that when neural networks (NN) were used, all authors would comment on the data input to decide whether it was used as a supervised or unsupervised learning algorithm. The chosen class would get +1 because it would depend on the task the NN is applied to. The term other refers to anything that was outside of the ML tasks such as Arden syntax, fuzzy expert map, and rule-based systems, which all occurred only once. The most frequent type of learning was supervised learning with 47 articles. Captivatingly, the number of studies mentioning reinforcement learning was 0.

**TABLE 5:** Studies split up per learning category and the most used algorithm per category

Type of learning	Study ID	Top used algorithms
Supervised	Yalçın et al., 2023 [28]; Timsina et al., 2020 [25]; Yin et al., 2021 [26,29]; Nel et al., 2022 [30]; Kiss et al., 2023 [31]; Wang et al., 2023 [32]; Momand et al., 2022 [33]; Khan and Yunus, 2023 [34]; Di Martino et al., 2021 [35]; Aryuni et al., [36]; Usman and Kopczewska 2022 [37]; Checchi et al., 2022 [38]; Ren et al., 2022 [39]; Sparapani et al., 2022 [40]; Islam et al., 2022 [41]; Kiss et al., 2022 [42]; Bitew et al., 2022 [43]; Fenta et al., 2021 [44]; Rahman et al., 2021 [45]; Vasu et al., 2021 [46]; Talukder and Ahammed, 2020 [47]; How and Chan, 2020 [48]; Kraamwinkel et al., 2019 [49]; Ohyver et al., 2017 [50]; Khare et al., 2017 [51]; Schüttler et al., 2017 [52]; Henrique et al., 2020 [53]; Ganju et al., 2021 [54]; Khudri et al., 2023 [55]; Browne et al., 2021 [56]; Tagi et al., 2022 [57]; Ren et al., 2023 [58]; Maasthi et al., 2023 [59]; Siwathammarat et al., 2023 [60]; Jin et al., [61], 2022; Sujatha et al., 2021 [62]	RF, LR, NN, DT, SVM

Unsupervised	Yin et al., 2021 [26]; Nel et al., 2022 [30]; Dhanamjayulu et al., 2022 [63]; Tagi et al., 2022 [57]; Siwathammarat et al., 2023 [60]; Yin et al., 2021 [64]; Bondi et al., 2022 [65]	NN

Semisupervized	How and Chan, 2020 [48]	—

Reinforcement learning	—	—

Qualitative sudy	Kirk et al., 2022 [27]; Sharma et al., 2020 [66]; Besculides et al., 2023 [67]; Paulsen et al., 2021 [68] Paulsen et al., 2019 [69]; Tay et al., 2022 [70]; Wang et al., 2022 [71]	—

Other	Petrauskas et al., 2021 [72]; de Bruin et al., 2018 [73]	Arden Syntax, Ranker algorithm

Abbreviations: DT, decision tree; LR, logistic regression; NN, neural network; RF, random forest, SVM, support vector machine.

#### What types of algorithms are being used?

When designing an optimal DSS, it is very important to not only investigate the type of learning that was used but also the type of algorithms that were used to execute the task. Every algorithm has its own pros and cons and should always be judged with context in mind. Important to note is that many primary studies used multiple algorithms either in subsequent order or to compare them with each other. Whenever an algorithm was used, it was denoted as +1 in assigned column (e.g., random forest is compared with logistic regression, both would get +1). The cumulative for all algorithms used therefore exceeds the total amount of primary studies. Finally, all forms of decision trees were added together for ease of interpretation.

**FIGURE 11:**
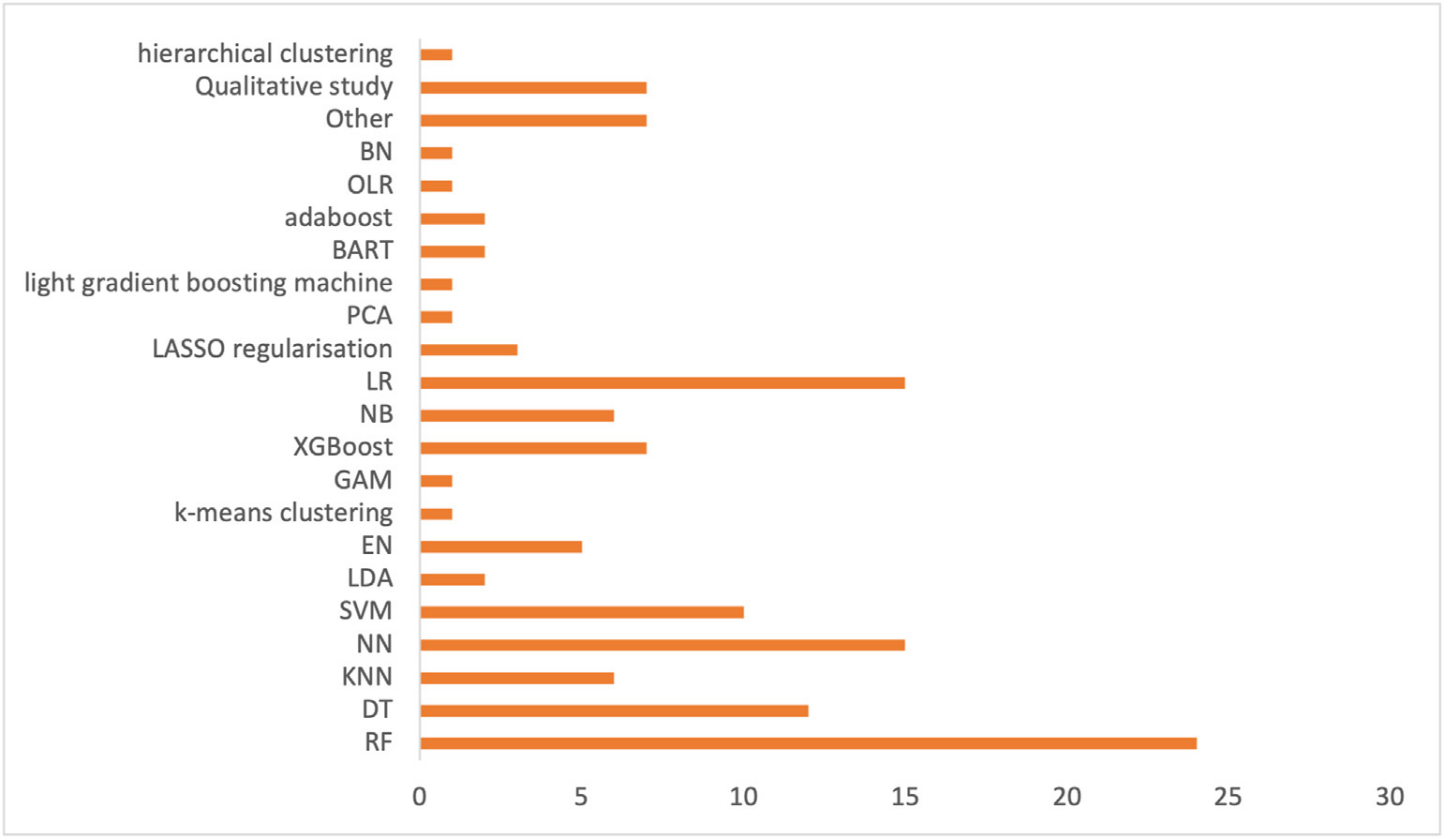
Types of algorithms used. BART, Bayesian additive regression tree; BN, Bayesian network; DT, decision tree; EN, elastic net regularization; GAM, generalized additive model; KNN, K-nearest neighbor; LASSO, least absolute shrinkage and selection operator; LDA, linear discriminant analysis; LR, logistic regression; NB, naive Bayes; NN, neural network; OLR, ordinary linear regression; PCA, principal component analysis; RF, random forest; SVM, support vector machine; XGBoost, extreme gradient boosting.

Figure 11 shows the types of algorithms that were used. Random forest was the most popular algorithm, used in 24 articles. From the graph, it can be seen that many algorithms were only used once. The algorithm with the least uses were hierarchical clustering, Bayesian network, ordinary linear regression, light gradient boosting, principal component analysis, generalized additive models, and k-means clustering, with 1 article for each algorithm.

#### What problems are the algorithms assigned to?

There are a handful of different tasks that exist. Some of these include classification, regression, dimensionality reduction, clustering, anomaly detection, and recommendation [27]. This study focused on regression and classification. Originally, all forms were included but no cases were found for anything except classification and regression. Only few articles used dimensionality reduction as a data preprocessing step. Classification refers to assigning a group label to data points to sort the data into different groups based on specific features. Regression refers to trend-based forecasting, for example, predicting male bodyweight based on the bodyweight of 10 previously measured males.

**FIGURE 12:**
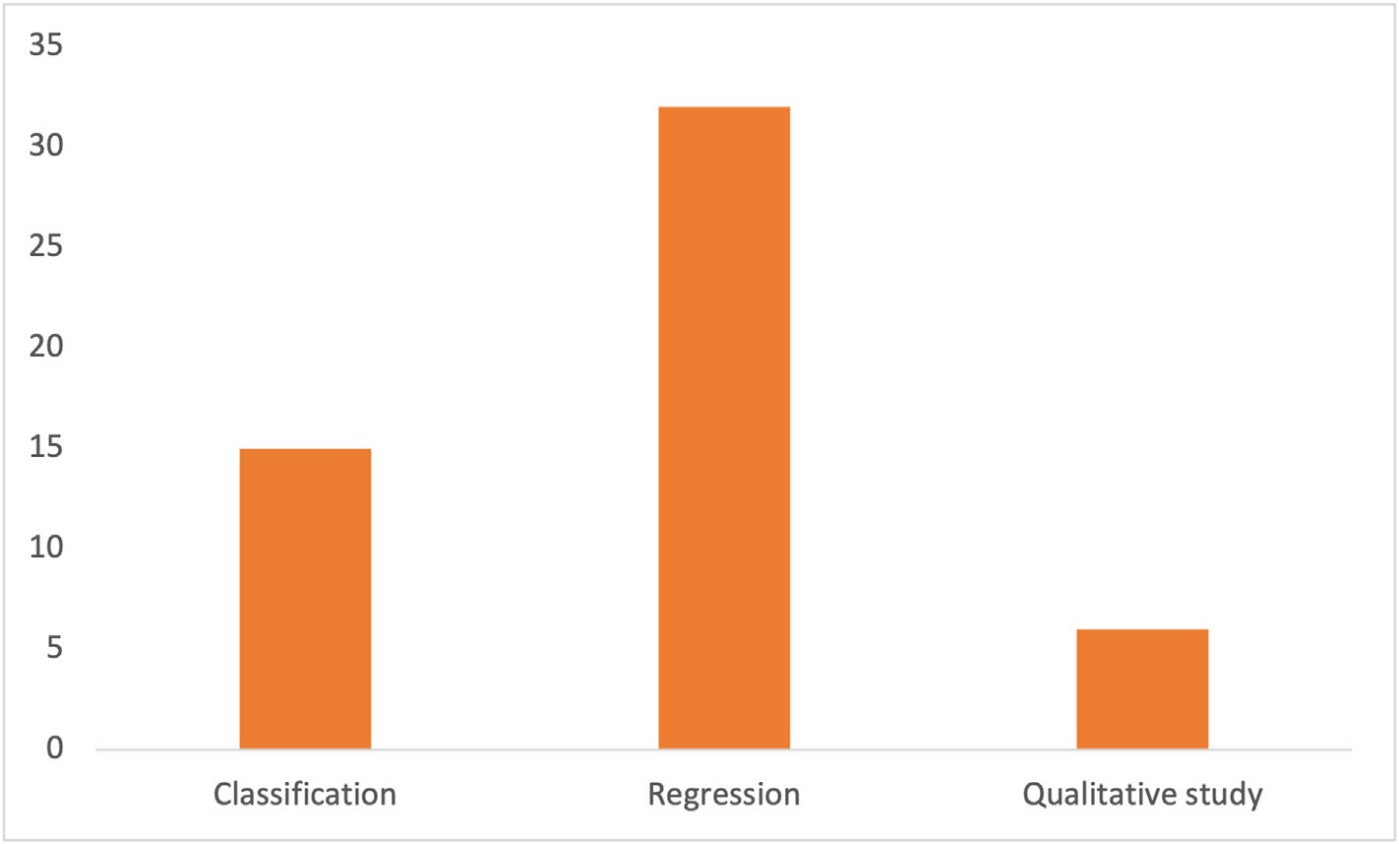
Types of tasks the algorithms were assigned to.

Regression was the most common type of task, used in 32 studies. Six studies were qualitative, and 1 study did not specific anything about a task. It was not possible to extract this information from the context. Classification was used in 15 times. The total count is depicted in Figure 12.

The next part strived to create a resource for those who plan on building their own models to apply regression or classify malnutrition in the future. To build good models to use for a DSS, the best input variables need to be selected. To do this, the primary studies were used as references to determine the most used variables in literature. Over 100 different variables were identified throughout the 49 primary studies. Several variables, such as reason for hospitalization and cancer site, encompass several subcategories or specific details like the cancer site. To give a trustworthy estimate, these are all enumerated in the top 10 as a newly created category (Table 6). This was done by hand to avoid missing any relevant data. Any variables that were very similar were added together. For instance, “age in years,” “age in months,” “age in days,” and “months since birth” would all be condensed into “age” because they mean the same thing. It must be stated that in doing so, some minor details may be lost in return for the readers’ convenience.

**TABLE 6:** Top 10 most used variables split by type of malnutrition

Type of malnutrition	Top 10 most used variables for model building
Disease-related malnutrition	Weight, BMI, age, serum albumin, serum creatinine, hemoglobin, serum sodium, blood urea nitrogen, serum potassium, platelets

Nondisease-related malnutrition (adult)	Age, residence, region, education, currently breastfeeding, currently working, marital status, currently pregnant, children ever born, partner’s education

Nondisease-related malnutrition (child)	Age; sex/gender; height-for-age < −2 SD; wasting weight-for-height < −2 SD and underweight; weight-for-age < −2 SD of the WHO Child Growth Standards median + 1; BMI; weight; height; birth weight; gestational age; mother age; mother education (none, primary, secondary, or higher)

Abbreviation: BMI, body mass index; SD, standard deviation; WHO, World Health Organization.

Finally, note that for the variables that have the same number of occurrences, the order of finding them was used. For example, weight is used 3 times, “BMI” and “age” are both used twice. In this case, “BMI” is put first because the article that was checked first had “BMI” and not “age.” This needs to be kept in mind when building the final model, and variable selection on your specific data should be done first.

### RQ 3: What is the stage of implementation?

This research question is used to gage the state of implantation of the current DSS in the literature. Many models are developed and look wonderful in theory, however, might not work optimally or the way they are supposed to in practice.

**FIGURE 13:**
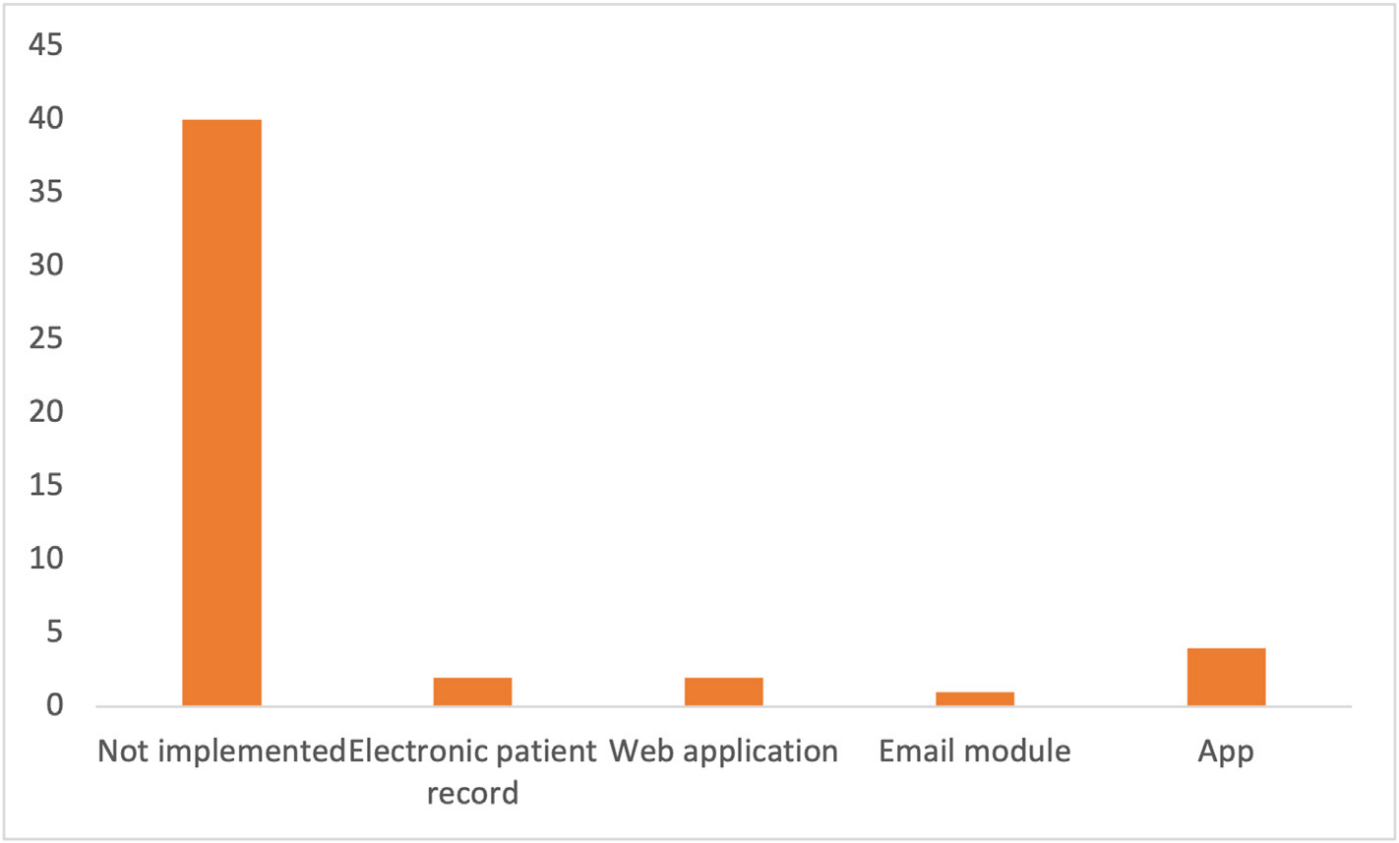
Implementation type count.

Every primary study was carefully read in its entirety to look for any text regarding implementation. Most studies (i.e., 41 articles) mentioned the implementation did not mention the implementation phase of the DSS (Figure 13). This is a surprisingly large number given its importance. Applications were the most popular implementation method, observed in 4 articles. One primary study used a built-in e-mail module to e-mail the outcome to a desired person [63]. Two web applications and electronic patient record implementations were built.

### RQ 4: What are the challenges when using DSS in malnutrition?

All primary studies were read in their entirety after which all mentioned limitations and challenges were extracted and categorized. This resulted in 37 different limitations and challenges, which were divided over 5 categories: model, study design, sample size, representation, and data set. Some of the limitations are highlighted in Table 7 [[25], [26], [27], [28], [29], [30], [31], [32], [33], [34],^36^,[38], [39], [40],[42], [43], [44], [45],^48^,^49^,^53^,[55], [56], [57], [58],^60^,^61^,^63^,^64^,[67], [68], [69], [70], [71],^73^]. The other primary studies did not mention any limitations.

**TABLE 7:** Limitations of primary studies sorted per category with their study ID

Category	Example of limitations per category	Study ID per category
Model	- There is an apparent overoptimism in ML research that exists owing to nonrigorous methodologies. - Better algorithms or predictors in the model like boosting could have been used or using least absolute shrinkage and selection operator to get better predictors could have been used.	Kirk et al., 2022 [27]; Yin et al., 2021 [26]; Checchi et al., 2022 [38]; Sparapani et al., 2022 [40]; Kiss et al., 2022 [42]; Fenta et al., 2021 [44]; Rahman et al., 2021 [45]; Kraamwinkel et al., 2019 [49]; Browne et al., 2021 [56]; Tagi et al., 2022 [57]; Ren et al., 2023 [58]; Yin et al., 2021 [64]; de Bruin et al., 2018 [73]; Dhanamjayulu et al., 2022 [63]; Aryuni et al., [36]; Tay et al., 2022 [70]; Bitew et al., 2022 [43]; How and Chan, 2020 [48]; Wang et al., 2022 [71]; Siwathammarat et al., 2023 [60]; Jin et al., 2022 [61]

Study design	- Retrospective study design and secondary analysis of the data, which meant that some variables, such as cancer stage, and longer-term outcomes that may be important to consider were not available. - Because this study is retrospective, no muscle mass data are available.	Yalçın et al., 2023 [28]; Timsina et al., 2020 [25]; Yin et al., 2021 [29]; Besculides et al., 2023 [67]; Nel et al., 2022 [30]; Kiss et al., 2023 [31]; Wang et al., 2023 [32]; de Bruin et al., 2018 [73]; Paulsen et al., 2019 [69]; Paulsen et al. 2021 [68]; Khan and Yunus, 2023 [34]; Ren et al., 2022 [39]; Henrique et al., 2020 [53]

Data set	- We did not have data on the clinical observations regarding the inflammation status, red cell distribution, or chronic use of steroids, which might have led to an underestimation of the number of patients with inflammation. - Training and test sets were derived from the same study, meaning the model needs to be validated by prospective research. Finally, there was a slight imbalance in the data, which resulted in lower precision because there was a low number of people with malnutrition.	Yin et al., 2021 [29]; Wang et al., 2023 [32]; Momand et al., 2022 [33]; Khan and Yunus, 2023 [34]; Jin et al., 2022 [61]; Rahman et al., 2021 [45]; Henrique et al., 2020 [53]; Khudri et al., 2023 [55]

Representation	- The availability of a wide range of data may not be uniform in different hospital settings.	Timsina et al., 2020 [25]; de Bruin et al., 2018 [73]

Sample size	- Small sample size	Yalçın et al., 2023 [28]; Dhanamjayulu et al., 2022 [63]; Henrique et al., 2020 [53]

The model category represents the limitations and challenges that have to do with the building, interpretation or use of the ML model that was constructed. This is the biggest category with 21 primary studies allocated to it. One primary study mentioned the fact that the model could benefit from potentially better predictors, which could be identified using least absolute shrinkage and selection operator regression [38]. Another review mentioned that there is an apparent overoptimization in ML research that exists owing to nonrigorous testing [27].

The study design category represents all limitations and challenges that have to do with problems that could be addressed by changing the study layout. A total of 13 studies were allocated to this category. Multiple studies mentioned the fact that the use of a retrospective design limited their data availability because of not having access to current biomarker values or time series data [29,31,39,58].

The data set category represents the limitations and challenges that related to data-related issues. In total, 8 primary studies were allocated to this category. For instance, 1 primary study mentioned that their test and training data set were derived from the same study, which means that the model might perform better than if it were to be tested on never seen before data [32].

Two studies mentioned problems relating to the representation of their research. One of such discussed the problem of their research using many different data sets to build their model. However, this may not be possible in other hospitals, which means their model would be simpler and perform worse. In this sense, their model shows a very optimistic result [25].

Finally, the sample size category comprised all studies that mentioned their challenges related to a small sample size, which may interfere with their results. In total, 3 studies were allocated to this category.

## Discussion

The following sections will be used to go over the discussion part of the present study. We aim to reflect on the results and relate it to all other relevant work and finally examine the threats to the validity of this study.

### Reflection on the results

To our knowledge, this study represents the first SLR on DSS in malnutrition and thereby aims to pave the way for similar studies on DSS in malnutrition. A search was conducted, and over 300 articles were identified of which 49 primary studies were included in the final analysis. Each article received a quality assessment score.

The first interesting finding is that the focus of the research being conducted depends on the country of origin and its development. Figure 5 depicts the spread of published primary studies. Research groups from more developed countries tend to focus more on disease-related malnutrition whereas developing countries focused on nondisease-related malnutrition. This may be due to the urgency of the problem. When there is no food present for people to eat, the prevalence of acute malnutrition is many times higher than that in developed countries where there is an abundance of food. Another example would be crisis-stricken countries that do not have the luxury of food abundance, so changes to body composition lasting from overeating do not occur as often as acute malnutrition [38]. This makes it so that these research groups focus on the most pressing issues in their direct environment. The developed countries focus on the body composition changes leading to disease-related malnutrition, and the developing countries tend to focus on non–disease-related malnutrition, which is mostly related to food shortages.

Second, after all used malnutrition screening tools were identified, a gap between practice and the literature was identified. Only a few malnutrition tools were identified in literature although there are plenty of others available in practice as highlighted in “RQ 1: What type of malnutrition tool is being used*?*” section. Arguably, the most important ones are covered in this SLR. However, all the ones specified in the literature relate to general purpose questionnaires, meaning they are designed for all patient groups. There are several other tools created for specific patient groups, which were not used in the literature. Moreover, some universal tools like the NRS-2002 [74] were not identified in the literature. Interestingly, this gap between practice and the present literature presents opportunities to conduct experiments with these other tools and compare them with the tools used up until this point in the literature. An important aspect of patient-specific tools is the lack of generalizability. Many of the tools and models reviewed are specific to certain patient groups or types of malnutrition, which limits their broader applicability across diverse clinical settings or geographical regions. This demonstrate a need for conducting research on a broader subgroup of malnutrition population.

One of the intriguing things that stood out was the large number of supervised models that were used in the included primary studies; 49 primary studies were identified of which 40 made use of supervised models. Notably, zero studies were identified with either semisupervized learning or reinforcement learning. Reinforcement learning is a very promising ML technique that offers different benefits from all other forms of learning. This type of learning provides unique abilities that may be of use to a good DSS like the ability to learn feedback in the form of a reward or penalty. Semisupervized techniques like transfer learning may be of use to create good models with limited amounts of labeled data, saving not only time but also money by removing the need for manual annotation of data labels.

Finally, the importance of the implementation stage of a DSS was accentuated. The results showed that 9 of 10 presented solutions were not implemented. This is a vital part of designing a DSS because the DSS can be improved after it is fed new data by using techniques like reinforcement learning. To overcome future challenges, DSS needs to be used in practice to discover any existing issues or weaknesses to this system. This can in turn be used to improve DSS in the future. The current literature lacks adequate representation of this iterative process, offering significant potential for future research. Further, the lack of implementation is indicating a significant gap between model development and practical deployment, which may undermine the impact of these innovations. In the future, more research should be done. Some studies included in this review have already started to identify practical steps that allow for deployment of these models [15,68,69].

### Related work

In recent years, the idea of using DSS of diagnosing or aiding in the treatment of malnutrition has become more popular. However, there is limited evidence of it being used and stated so in literature. Some studies have considered the use of DSS for diagnostic purposes to increase patient health outcomes. One of such studies is a SLR by Vasey et al. [75] on the association of clinician diagnostic performance with ML-based DSSs. This is a very well-designed study but does not cover the topic of malnutrition. Other articles have been published with regards to DSS based on ML but either they did not cover the topic of malnutrition or they were related to an intervention program using DSSs. Some scientists have used DSS as aid in treating malnutrition but not based on ML but based on existing guidelines and traditional tools [76]. Moreover, some intervention studies have been conducted about the role of ML in DSS [16,67]. However, this does not translate to specific research on malnutrition. The related work identified multiple use cases of DSSs in health care based on either existing guidelines or ML.

The main difference between this study and the related work is the use of the SLR format. This allows for discovery of information after which an overview was created. This in turn can be used to identify research gaps, which otherwise would go unnoticed. For example, we identified zero of 49 studies that made use reinforcement learning. This approach to DSS may yield new and useful results. It seems that many of the existing solutions presented in the literature are not implemented into daily practice. This is an important step to improve the health care system, which is the final goal. Interestingly, many screening tools for malnutrition screening tools like SNAQ, NRS-2002, or Strongkids are used in practice but were not documented a single time in the literature. This means that there is a gap between current tool use in practice and screening tools documented in the literature. This might be because journal articles were used and no gray literature. Including gray literature could change the outcome.

### Challenges and opportunities of implementation

This section highlights challenges in integration, acceptance, and implementation of DSSs within the health care system despite their potential in clinical nutrition. First, increasing transparency of ML models used should be one of the key components of a well-designed model. One way is to use simulations to empirically evaluate the variability of model metrics and explanatory algorithms to observe whether covariates match the literature are necessary for increased transparency, reliability, and utility of ML methods [77]. This can be done with bootstrapping simulations to quantify the statistical distributions of model accuracy metrics. Simulations of these distributions have been proven to work well in a cross-study comparison setting in which model evaluation metrics need to be compared [78,79]. A great opportunity within ML research lies in the fact that ML models can be used to assess risk factors for a given disease. For instance, Huang and Huang [80] used ML to identify risk factors for insomnia, which provides insights into how similar methodologies can be adapted for identifying risk factors associated with malnutrition, thereby aiding in early diagnosis and intervention. Solving these challenges may prove to be worthwhile as it will allow for ML models to become more widespread in use as well as be able to fit in various clinical workflows. Being able to not only compare models across different clinical use cases but also improve transparency will allow for easy integration into various health care systems.

Second, many experts throughout different fields voiced their concerns regarding the need for rules and new policies in the field of AI. Ethics is one of the most relevant aspects that should at all-time be kept in our minds as we progress toward mainstream AI usage. Ethical and regulatory challenges posed by the novelty of AI systems in health care such as bias, data protection, or explainability must be addressed by states or regulatory bodies [81,82]. One of the driving factors behind this change is that AI is expected to play a major role in achieving the sustainable development goals of the United Nations [83]. As we go on to create more advanced AI systems and strive to integrate these in many aspects of our lives, it is of utmost importance to involve many different disciplines. In health care, not only physicians but also policy makers, public health experts, software engineers, and legal experts should be involved to create coherent policies or rules regarding the use of these new technologies [84,85]. In addition, many scientists stress the importance of more research in the field of the ethics of AI in health care [81,86].

Finally, resource allocation and training costs are an important aspect to accelerate the AI adoption. A scoping review was conducted to determine economic impact of AI in health care [87]. The authors conclude that more comprehensive economic assessments should be done to enable economic decisions for or against AI use. Moreover, medical professionals need comprehensive training in the use of AI tool to develop a cohesive relationship between AI and human efforts to improve patient care. This can be done by investing time and resources into training programs and the implementation phase of AI [88].

### Addressing threats to validity

Every type of study design has its own pros and cons when it comes to validity. The SLR is prone to publication and selection bias as it uses data extraction and classification [89]. To address this threat of publication bias, the quality assessment score was used. This allowed for exclusion of studies that did not adhere to this quality threshold of ≥4 of 8 points. This resulted in the exclusion of 5 low-quality primary studies, which did not present any limitations. Second, the threat of selection bias refers to the situation in which the SLR fails to address all available data on a topic [89]. To address these, 4 different digital libraries were used (Table 1) until we noticed that many of the results were the same articles. Next to this, a manual search was conducted by using the snowballing and backward snowballing techniques. Exclusion criteria were established to remove any irrelevant articles (Table 2) to maintain only the relevant data. These criteria were discussed among all coauthors to ensure their quality. The importance of the validity of data extraction is of utmost importance as it directly influences the results of this SLR. To ensure the quality of the extraction process, 1 coauthor gave a second opinion on ≥15 articles. All articles that were not clearly fit for inclusion or exclusion were discussed with the other coauthors. Third, owing to the complexity of the topic of malnutrition, there is a risk that data synthesis caused information to be lost because of the limitation of available categories. The data synthesis was performed with the end goal of keeping as much distinction as possible between the concepts as possible. When in doubt, the original article was referenced to categorize the data. Finally, 49 high-quality primary studies were included. The possibility that some quality articles were missing will always remain; however, we believe we were able to identify a reasonable amount of input data for this SLR. Applying the aforementioned measure, we believe we have tackled the main threats to the validity.

## Conclusions

In this study, an SLR was executed in the scientific literature of the past 5 y to identify the current state of DSS for malnutrition detection and classification using ML. To our knowledge, this is the first SLR on this topic and, therefore, aims to pave the way for all upcoming research related to DSS in malnutrition. The SLR approach allowed for useful data extraction and visualization. This led to novel insights into the current state of literature on DSS in malnutrition. First, we used bibliometric networks to visualize the most used keywords and trends of these keywords throughout the last 5 y. This visualization allowed for a deeper understanding of the current trends in literature. We identified that the current research on this topic is done roughly half of the time in non–disease-related malnutrition often relating to children and the other part is relating to disease-related malnutrition of institutionalized adults in the clinical setting. The first was mostly conducted in poorer less developed countries and the latter in more developed countries. In both, the most popular use case for DSS using ML was supervised learning algorithms that were mostly used for regression tasks. Other forms of learning such as reinforcement learning were absent in all included articles. We were able to identify the 10 most used variables for all included patient groups as well as identify the most used ML algorithms. Then, we highlighted the fact that over 90% of all created solutions have not been implemented in practice, underlining the apparent gap between practice and the current state of the literature on DSS in malnutrition. Furthermore, we were able to highlight the most encountered challenges in the present literature. The challenges we identified were not aimed to criticize the existing DSS but rather to pave the way for further maturation of these DSSs. Future studies can be executed to investigate the implementation of these DSS or other forms of learning such as reinforcement learning. In summary, practitioners can benefit from the results of this study by a thorough knowledge of potential challenges along the road of creating the best possible DSS for malnutrition.

## Author contributions

The authors’ responsibilities were as follows – SMWJ: was responsible for conceptualization, methodology, writing—original draft, review, and editing; YB, BT: were responsible for conceptualization, methodology, supervision, review, and editing; and all authors: read and approved the final manuscript.

## Conflict of interest

The authors report no conflicts of interest.

## Funding

The authors reported no funding received for this study.

## Data availability

The data that support the findings of this study are available on request from the corresponding author, Y. Bouzembrak.

## Footnotes

1 https://www.vosviewer.com
