# Supplementary material for: Artificial Intelligence in Malnutrition: A Systematic Literature Review
Source: Adv Nutr. 2024 Jul 4;15(9):100264. doi: 10.1016/j.advnut.2024.100264 (PMC11403436; doi:10.1016/j.advnut.2024.100264)
Supplement: Multimedia component 1 [file mmc1.docx]

**Artificial Intelligence in Malnutrition: A systematic literature review**

Janssen et al. (2024)

**Table A.1:** All search queries by database.

| PubMed | ("Malnutrition"[Title/Abstract] OR "Undernutrition"[Title/Abstract]) AND ("Tool*"[Title/Abstract] OR "app*"[Title/Abstract]) AND ("Artificial Intelligence"[Title/Abstract] OR "Machine Learning"[Title/Abstract]) |
| --- | --- |
| Scopus | (TITLE-ABS-KEY ( malnutrition ) OR TITLE-ABS-KEY ( undernutrition ) ) AND ( TITLE-ABS-KEY ( tool* ) OR TITLE-ABS-KEY ( app* ) ) AND ( TITLE-ABS-KEY ( "Artificial intelligence" ) OR TITLE-ABS-KEY ( "Machine Learning" ) ) AND PUBYEAR > 2012 AND PUBYEAR < 2024 |
| Web of Science | TS=("Malnutrition" OR "Undernutrition") AND TS=("Tool*" OR "app*") AND TS=("Artificial Intelligence" OR "Machine Learning") |
| Google Scholar | allintitle: Malnutrition machine learning  allintitle: Malnutrition decision support  allintitle: malnutrition app |

**Table A.2:** The 49 primary studies used as input for the SLR and the corresponding publication year.

| *ID* | *Year* | *ID* | *Year* | *ID* | *Year* |
| --- | --- | --- | --- | --- | --- |
| (Sharma et al. 2020) | 2020 | (Di Martino, Delmastro, and Dolciotti 2021) | 2021 | (Kraamwinkel et al. 2019) | 2019 |
| (Kirk et al. 2022) | 2022 | (Usman and Kopczewska 2022) | 2022 | (Ganju et al. 2021) | 2021 |
| (Yalçın et al. 2023) | 2023 | (Checchi et al. 2022) | 2022 | (Khare et al. 2017) | 2017 |
| (Timsina et al. 2020) | 2020 | (Ren et al. 2022) | 2022 | (Schüttler et al. 2017) | 2017 |
| (Yin, Lin, et al. 2021) | 2021 | (Sparapani et al. 2022) | 2022 | (Ohyver et al. 2017) | 2017 |
| (Yin, Song, et al. 2021) | 2021 | (Jin et al. 2022) | 2022 | (Khudri et al. 2023) | 2023 |
| (Besculides et al. 2023) | 2023 | (Bondi et al. 2022) | 2022 | (Browne et al. 2021) | 2021 |
| (Nel et al. 2022) | 2022 | (Tay et al. 2022) | 2022 | (Tagi et al. 2022) | 2022 |
| (Kiss et al. 2023) | 2023 | (Islam et al. 2022) | 2022 | (W. Wang et al. 2022) | 2022 |
| (X. Wang et al. 2023) | 2023 | (Kiss et al. 2022) | 2022 | (Maasthi et al. 2023) | 2023 |
| (Momand, Zarinkhail, and Aryan 2022) | 2022 | (Bitew, Sparks, and Nyarko 2022) | 2022 | (Ren et al. 2023) | 2023 |
| (Petrauskas et al. 2021) | 2021 | (Fenta, Zewotir, and Muluneh 2021) | 2021 | (Siwathammarat, Jesadaporn, and Chawachat 2023) | 2023 |
| (de Bruin et al. 2018) | 2018 | (Rahman et al. 2021) | 2021 | (Yin, Liu, et al. 2021) | 2021 |
| (Dhanamjayulu et al. 2022) | 2022 | (Vasu et al. 2021) | 2021 | (K. Sujatha et al. 2021) | 2021 |
| (Paulsen et al. 2019) | 2019 | (Talukder and Ahammed 2020) | 2020 | (Aryuni et al.) | 2022 |
| (Paulsen, Varsi, and Andersen 2021) | 2021 | (How and Chan 2020) | 2020 |  |  |
| (Khan and Yunus 2023) | 2023 | (Henrique et al. 2020) | 2020 |  |  |
